# Acidic urine is associated with poor prognosis in patients with bladder cancer undergoing radical cystectomy

**DOI:** 10.3389/fonc.2022.964571

**Published:** 2022-08-26

**Authors:** Jang Hee Han, Seung-hwan Jeong, Hyeong Dong Yuk, Chang Wook Jeong, Cheol Kwak, Ja Hyeon Ku

**Affiliations:** ^1^ Department of Urology, Seoul National University Hospital, Seoul, South Korea; ^2^ Department of Urology, Seoul National University College of Medicine, Seoul, South Korea

**Keywords:** urine pH, acidic, bladder cancer, recurrence, survival

## Abstract

**Purpose:**

To assess the prognostic value of acidic urine (low urine pH) in patients with bladder cancer undergoing radical cystectomy.

**Materials and methods:**

We reviewed patients enrolled in the Seoul National University Prospectively Enrolled Registry for Urothelial Cancer-Cystectomy (SUPER-UC-Cx) who underwent radical cystectomy for bladder cancer between March 2016 and December 2020 at the Seoul National University Hospital. During this period, 368 patients were registered in our database. To eliminate confounding factors, we excluded patients diagnosed with non-urothelial cancer and end-stage renal disease.

**Results:**

A total of 351 patients with a mean age of 69.8 ± 10.5 years and median follow-up of 16.0 months were eligible for the analysis. The mean preoperative urine pH was 6.0. The patients were divided into low (pH ≤ 5.5) and high (pH≥6.0) urine pH groups for comparison. All clinicopathological features, including the tumor size, grade, and stage were comparable between the low and high urine pH groups. A Cox regression analysis was performed to assess the independent effect of acidic urine on patient survival. A multivariate analysis showed that high T stage (T3-4) (hazard ratio (HR) 5.18, *P*<0.001), decreased renal function (estimated glomerular filtration rate <60 mL/min/1.73 m^2^) (HR 2.29, *P*=0.003), and low urine pH (≤5.5) (HR 1.69, *P*=0.05) were associated with shortened recurrence-free survival (RFS). Regarding the overall survival (OS), high T stage (T3-4) (HR 7.15, *P*<0.001) and low urine pH (≤5.5) (HR 2.66, *P*=0.029) were significantly associated with shortened survival. A Kaplan–Meier analysis demonstrated that the acidic urine group showed shorter RFS (*P*=0.04) and OS (*P*=0.028) than the other groups.

**Conclusions:**

Acidic urine was independently associated with reduced RFS and OS in patients with bladder cancer undergoing radical cystectomy. Acidic urine contributing to an acidic tumor environment may promote aggressive behavior in bladder cancer.

## Introduction

Bladder cancer is the 10th most prevalent cancer worldwide, with approximately 550,000 new cases diagnosed each year  (1, 2), and its incidence is continuously rising. While the five-year survival rate of localized bladder cancer is approximately 70%, it rapidly decreases as the cancer extends to a locally advanced stage, with a five-year survival rate of 38% (3). Accordingly, radical cystectomy is now expanding its indication as the term “early cystectomy” for better oncological outcomes. Indeed, several studies have proven the survival benefits of this approach (4, 5). Moreover, to obtain a better solution for this poor prognostic cancer, studies have focused on the identification and stratification of risk factors *via* orthogonal approaches, including genetic analysis and molecular subtyping, to enhance the survival outcomes (6–9).

Similar to other tumors, bladder cancer also reprograms its metabolism to rely on aerobic glycolysis (10), resulting in lactic acid secretion and an acidic tumor microenvironment. Unlike normal cells that undergo cell death under acidic environmental exposure, tumor cells have the ability to adapt to chronic acidic conditions (11). This adaptation results in the cells acquiring invasiveness, stemness, and chemoresistance, all of which are correlated with tumor progression and poor overall survival (OS) (12–15). Indeed, these results are fully supported by previous studies that elucidate the role of extracellular acidic media in intracellular signaling leading to aggressive phenotypes (16).

Bladder cancer has a very distinct tumor environment, as approximately half of these tumors are exposed to urine on one side. As the urothelium is known to buffer acidic urine for reabsorption into the bloodstream (17), this proves that normal cells and bladder cancer cells lining the urothelium can be affected by the pH of the urine. Thus, we hypothesized that acidic urine may aggravate the acidity of the tumor environment, thereby contributing to invasive and intractable characteristics and resulting in a reduced recurrence-free survival (RFS) and OS. In this context, we assessed the prognostic value of preoperative acidic urine (low urine pH) in patients with bladder cancer undergoing radical cystectomy.

## Materials and methods

### Ethics approval and informed consent

This study was approved by the Institutional Review Board of the Seoul National University Hospital (IRB no. 2205-118-1327). The requirement for informed consent was waived owing to the retrospective study design. This study was performed in accordance with applicable laws and regulations, good clinical practice, and ethical principles, as described in the Declaration of Helsinki.

### Patient population

We reviewed the patients enrolled in the Seoul National University Prospectively Enrolled Registry for Urothelial Cancer-Cystectomy (SUPER-UC-Cx), who underwent radical cystectomy for bladder tumors between March 2016 and December 2020 at the Seoul National University Hospital. During this period, 368 patients were registered in the database. To eliminate the confounding factors, we excluded patients diagnosed with non-urothelial cancer and those with end-stage renal disease. Ultimately, 351 patients were included in the analysis.

### Measurement and definition of acidic urine

Urine pH was measured using a reagent test strip in which methyl red and bromothymol blue yielded different colors at different pH values. Using the test strip, the pH of midstream urine was determined based on the color in 30–60 s at 0.5 intervals. Acidic urine was defined as urine pH ≤5.5 using the dipstick test based on the previous definition by Bono et al., suggesting that the average urine pH was 6.0 (18, 19). The urine pH distribution of patients is depicted in **Supplementary Figure S1**.

### Definition of clinical albuminuria and its role as a confounding factor

A systemic review and meta-analysis defined clinically significant albuminuria as a dipstick test value of 2+ (albuminuria>30 ml/dl) (20). Accordingly, we used the same cutoff value for the clinical albuminuria classification. Clinical albuminuria (high burden of urine albumin) was considered as a confounding factor for several reasons. 1) The high burden of urine albumin may affect the urine pH owing to its weak acidic characteristics (21), 2) a high burden of urine albumin is complexly affected by the tumor and tumor stage (22) and renal function (23).

### Collected parameters

The demographic and clinicopathological data of patients, including sex, age at surgery, accompanying comorbidities, preoperative imaging findings, preoperative laboratory findings (serum creatinine, estimated glomerular filtration rate, and urine pH), operative findings (type of surgery, operation time, and estimated blood loss), pathology (size, histology, grade, stage, and carcinoma *in situ*, etc.), performance of neo-adjuvant/adjuvant chemotherapy, performance of Bacillus Calmette-Guerin (BCG), and oncological outcomes (RFS and OS) were collected.

### Statistical analysis

Differences in the clinical and pathological characteristics of patients with low pH (≤5.5) and high pH (>5.5) of urine were compared using independent Student’s t-test and chi-square test. Univariate and multivariate Cox regression analyses were performed to assess the independent influence of the possible risk factors on disease free survival and OS. All statistical analyses were performed using SPSS version 25 software (SPSS, version 25.0.0.2, IBM Corp., Armonk, NY, USA). A *P *value <0.05 was considered as statistically significant, and all the statistical tests were two-sided.

## Results

### Baseline characteristics

This study included 351 patients who underwent radical cystectomy due for bladder cancer. The mean age was 69.8 ± 10.5 years and male patients were predominant (n=275, 78.3%). Most of the patients manifested pathologically proven high grade bladder cancer (n=343, 93.2%). High T stage (T3-4) accounted for 33% of patients (**Table 1**). The mean urine pH was 6.0. Clinical albuminuria was found in 32% (n=113) of patients, and the median follow-up period was 16 months.

**Table 1:** Baseline characteristics.

Total (n)	351
Age (yrs) (mean ± SD)	69.8 ± 10.5
Sex (Male) (n, %)	275 (78.3)
BMI (kg/m^2^) (mean ± SD)	23.7 ± 3.7
HTN (n, %)	172 (49.0)
DM (n, %)	88 (25.1)
Previous BCG (n, %)	70 (19.9)
Previous TURB number (n, %)	
0-1	237 (67.5)
≥2	114 (32.5)
Tumor size (cm)	4.11 ± 3.30
Multiplicity (n, %)	10 (2.8)
Grade (n, %)	
High grade	343 (93.2)
Low grade	5 (1.4)
T stage (n, %)	
T1-2	232 (66.1)
T3-4	116 (33.0)
N stage (n, %)	
N0	267 (76.1)
N1-2	58 (16.5)
Lymph node density (%)	4.51 ± 13.9
Preoperative lab	
Creatinine (mg/dL)	1.18 ± 1.05
eGFR (mL/min/1.73 m^2^)	74.5 ± 26.9
Urine pH	6.06 ± 0.78
Urine albuminuria (n, %)	
0-1 positive (< 30 mg/dL)	237 (67.5)
≥2 positive (≥ 30 mg/dL)	113 (32.2)
Neo-adjuvant chemotherapy (n, %)	87 (24.8)
Adjuvant chemotherapy (n, %)	55 (15.7)
Median follow up period (months)	16.0 (5.9-24.8)
Median recurrence free survival (months)	12.4 (4.9-24.3)
Median overall survival (months)	15.8 (5.8-24.4)

BMI, body mass index; eGFR, estimated glomerular filtration rate.

### Perioperative characteristics

An open radical cystectomy was performed in 67% of the patients, while robotic radical cystectomy was performed for the rest of the patients (**Table 2**). Pelvic lymph node dissection was performed in >90% of patients. With regard to a pathological diagnosis, histologic variants were observed in 16% of patients, and among them, the squamous type was most common. Carcinoma *in situ* was observed in 121 patients (34.5%).

**Table 2:** Operative and pathological characteristics.

Total (n)	351
Type of surgery method (n, %)	
Open	236 (67.2)
Robotic	114 (32.5)
Type of diversion (n, %)	
Conduit	102 (29.1)
Neobladder	238 (67.8)
Pelvic lymph node dissection (n, %)	320 (91.2)
Operative time (minutes) (mean ± SD)	299 ± 321
Estimated blood loss (mL) (mean ± SD)	772 ± 684
Histologic variant (n, %)	56 (16.0)
Squamous type	25 (7.1)
Others	31 (8.9)
Lymphatic invasion (n, %)	77 (21.9)
Venous invasion (n, %)	27 (7.7)
Lymphovascular invasion (n, %)	83 (23.6)
Perineural invasion (n, %)	66 (18.8)
Seminal vesicle invasion (n, %)	15 (4.3)
Carcinoma *in situ* (n, %)	121 (34.5)

### Comparison of low and high urine pH groups

An analysis was performed for the patients who did not show clinical albuminuria (n=231). The number of patients in the low (≤5.5) and high (>5.5) urine pH groups was 115 and 116, respectively. The low (≤5.5) and high (>5.5) urine pH groups showed comparable clinical characteristics. Age, sex, BMI, and accompanying comorbidities (hypertension and diabetes mellitus) were comparable between the two groups. The incidence of smoking (*P*=0.316) and renal function were also comparable (estimated glomerular filtration rate, *P*=0.374). The numbers of patients who received neoadjuvant and adjuvant chemotherapy were similar (*P*=0.968 and 0.385, respectively). Disease recurrence (31.3% vs. 19.8%, *P*=0.046) and death events (13.3% vs. 5.2%, *P*=0.034) were observed to occur more in the low urine pH group (**Table 3**). Regarding the pathological characteristics, the tumor size (*P*=0.092), T stage (*P*=0.485), N stage (*P*=0.476), lymph node density (*P*=0.238), carcinoma *in situ* (*P*=0.618), and histologic variants (*P*=0.189) were all comparable between the two groups (**Table 4**).

**Table 3:** Clinical characteristics of low and high urine pH groups.

	Urine pH ≤5.5 (n=115)	Urine pH >5.5 (n=116)	*P*-value
Age (years) (mean ± SD)	69.69 ± 10. 02	70.28 ± 9.89	0.653
Sex (Male) (n, %)	95 (82.6)	91 (78.4)	0.425
BMI (kg/m^2^) (mean ± SD)	24.06 ± 3.69	23.80 ± 3.08	0.564
HTN (n, %)	47 (40.9)	55 (47.4)	0.317
DM (n, %)	30 (26.1)	20 (17.2)	0.103
Smoker (n, %)			0.316
Ever smoker	69 (60)	61 (52.6)	
Never smoker	46 (40)	55 (47.4)	
Previous BCG (n, %)	26 (22.6)	31 (26.7)	0.468
re-TURB (n, %)	7 (6.1)	12 (10.3)	0.239
Creatinine (mg/dL)	1.04 ± 0.38	0.98 ± 0.33	0.259
eGFR (mL/min/1.73 m2)	76.09 ± 23.24	79.63 ± 25.83	0.374
Neo-adjuvant chemotherapy (n, %)	31 (27.0)	31 (26.7)	0.968
Adjuvant chemotherapy (n, %)	16 (13.9)	21 (18.1)	0.385
Recurrence (n, %)	36 (31.3)	23 (19.8)	0.046
Death (n, %)	15 (13.3)	6 (5.2)	0.034

eGFR, estimated glomerular filtration rate.

**Table 4:** Pathological characteristics of the low and high urine pH groups.

	Urine pH ≤5.5 (n=115)	Urine pH >5.5 (n=116)	*P*-value
Tumor size (cm) (mean ± SD)	3.73 ± 2.99	3.00 ± 2.24	0.092
Grade (n, %)			1.000
High grade	113 (98.3)	113 (97.4)	
Low grade	1 (0.9)	1 (0.9)	
T stage (n, %)			0.485
T1-2	74 (64.3)	79 (68.7)	
T3-4	41 (35.7)	36 (31.3)	
N stage (n, %)			0.476
N0	90 (81.8)	88 (85.4)	
N1-2	20 (57.1)	15 (14.6)	
Lymph node density (%)	5.29 ± 14.29	3.15 ± 12.05	0.238
Perineural invasion (n, %)	26 (22.6)	20 (17.2)	0.392
Carcinoma *in situ* (n, %)	39 (33.9)	44 (37.9)	0.618
Lymphovascular invasion (n, %)	27 (23.5)	23 (19.8)	0.607
Venous invasion (n, %)	9 (7.8)	9 (7.8)	1.000
Lymphatic invasion (n, %)	26 (22.6)	20 (17.2)	0.392
Histologic variant (n, %)	13 (11.3)	20 (17.2)	0.189

### Survival analysis predicting RFS and OS

**Figure 1:**
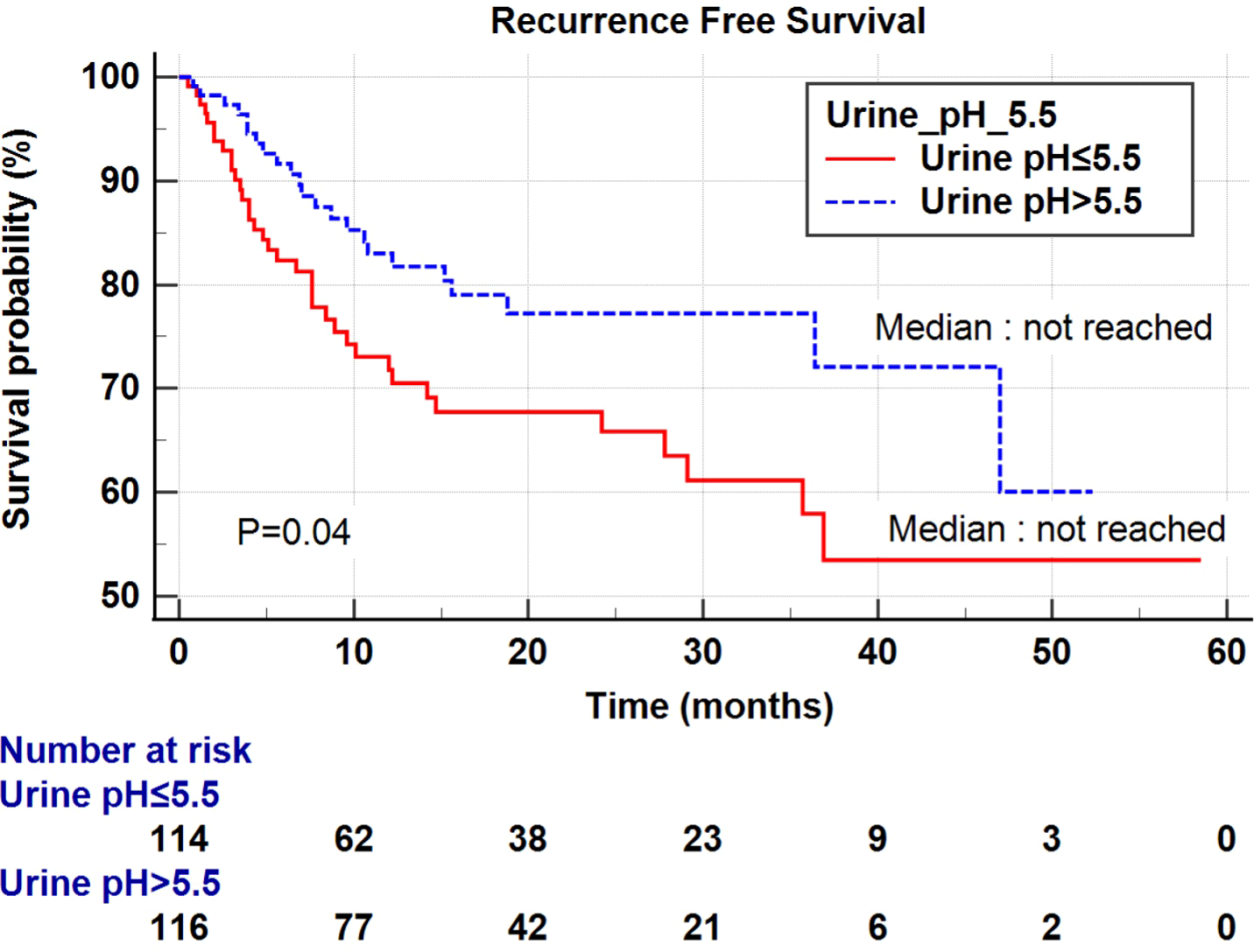
Kaplan–Meier curve of the effect of low urine pH (≤ 5.5) (red) and high urine pH (> 5.5) (dotted blue) groups on the recurrence-free survival.

**Figure 2:**
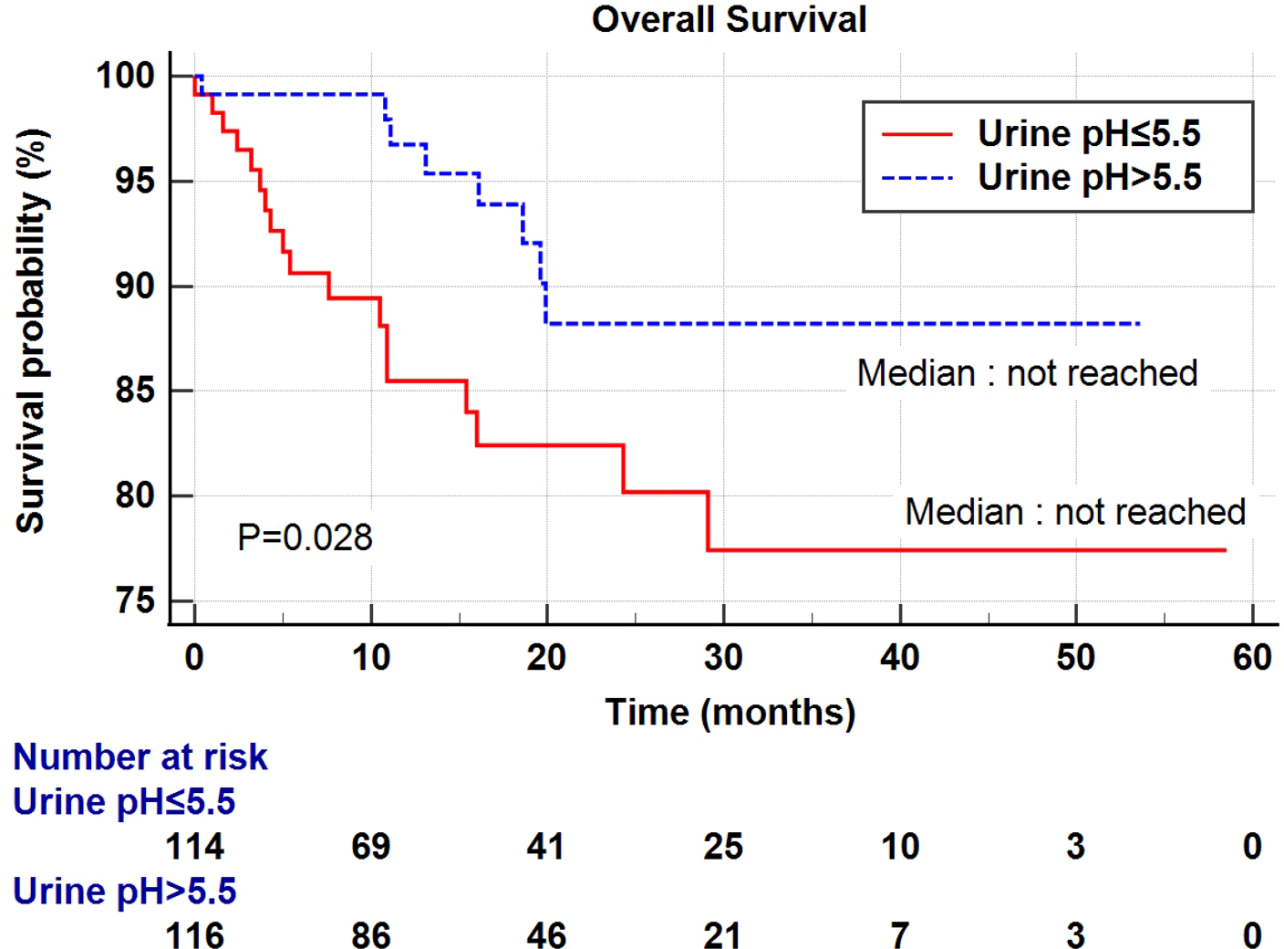
Kaplan–Meier curve of the effect of low urine pH (≤ 5.5) (red) and high urine pH (> 5.5) (dotted blue) groups on the overall survival.

Univariate and multivariate Cox regression analyses were performed to determine the factors affecting RFS and OS. A univariate analysis identified a higher T stage (T3-4), lower renal function (eGFR<60 mL/min/1.73 m^2^), and low urine pH (pH ≤ 5.5) as the significant risk factors for RFS (**Table 5**). In a multivariate analysis, the same risk factors were independently associated with a shortened RFS as follows: T stage 3-4 (hazard ratio (HR) 5.18, *P*<0.001), lowered renal function (eGFR<60 mL/min/1.73 m^2^) (HR 2.29, *P*=0.003), and low urine pH (pH ≤ 5.5) (HR 1.69, *P*=0.05). With regard to the OS, a higher T stage (T3-4), higher N stage (N1-2), and low urine pH (pH ≤ 5.5) were the significant risk factors in the univariate analysis. Among them, only a high T stage (T3-4) (HR 7.15, *P*<0.001) and low urine pH (pH ≤ 5.5) (HR 2.66, *P*=0.029) were significantly associated with shortened OS (**Table 5**). A Kaplan–Meier analysis was used to study the association of acidic urine or low urine pH (pH ≤ 5.5) with RFS and OS. The low urine pH group showed shortened RFS (*P*=0.04) (**Figure 1**) and OS (*P*=0.028) compared to the other groups (**Figure 2**).

**Table 5:** Cox regression analysis for disease recurrence and death.

	Univariate analysis			Multivariate analysis		
Variables	HR	95% CI	*P*-value	HR	95% CI	*P*-value
*Disease Free Survival*						
Age (>70) (yrs)	1.01	0.98-1.04	0.455			
Tumor size >4 cm	1.04	0.93-1.15	0.515			
Tumor stage (T3-4)	5.53	3.19-9.56	<0.001	5.18	2.98-8.99	<0.001
N stage (N1-2)	1.40	0.73-2.68	0.310			
Concomittant CIS	0.87	0.51-1.48	0.603			
eGFR<60 (mL/min/1.73 m^2^)	2.67	1.57-4.55	<0.001	2.29	1.33-3.93	0.003
Urine pH ≤ 5.5	1.72	1.02-2.90	0.043	1.69	1.00-2.86	0.050
*Overall Survival*						
Age (>70) (yrs)	1.59	0.71-3.56	0.264			
Tumor size >4 cm	2.18	0.96-5.00	0.064			
Tumor stage (T3-4)	7.60	3.05-18.9	<0.001	7.15	2.65-19.3	<0.001
N stage (N1-2)	3.16	1.39-7.15	0.006	1.08	0.45-2.62	0.862
Concomittant CIS	1.03	0.47-2.27	0.943			
eGFR<60 (mL/min/1.73 m^2^)	1.04	0.39-2.78	0.932			
Urine pH ≤ 5.5	2.47	1.07-5.69	0.034	2.66	1.11-6.40	0.029

eGFR, estimated glomerular filtration.

## Discussion

When tumor cells adapt to acidic environments, several concurrent cellular events occur, such as increased levels of acid-induced cell motility, extracellular matrix degradation, and invasion and metastasis (24, 25). Furthermore, extracellular acidity impairs the tumor response to anti-cancer drugs, such as classical platinum-based chemotherapy (26), as well as immunotherapy by suppressing the T cell-mediated immunity (27). As immunotherapy is emerging as an effective second-line treatment for metastatic urothelial cancer (28), an acidic tumor microenvironment may be a clinical hurdle that hinders the effective delivery of immunotherapy.

In this context, several ongoing studies are actively developing drugs that target the acidic tumor microenvironment for drug delivery or that modify the tumor microenvironment for effective treatment (29). For the formal approach, neutralizing the acidic environment using interfering pH-regulating systems, buffer therapy, or pH-sensitive drug-delivery system is widely being tried in numerous cancers, such as esophageal, colorectal, prostate, kidney, leukemia, and other solid tumors (11, 15). For the latter approach, a nanoparticle-based therapeutic approach is currently under investigation (30). Based on the clinical and prognostic significance of the acidic tumor microenvironment, we used clinical data to investigate whether acidic urine may further contribute to an acidic environment, resulting in shortened RFS and OS.

In fact, there have been several studies focusing on the relationship between urologic cancer and low urine pH. Ceylan et al. reported that acidic urine may be important for prostate cancer diagnosis, reporting low urine pH in prostate cancer patients compared to benign prostatic hyperplasia (31). Wright et al. reported that the relative risk of bladder cancer development increased in patients with acidic urine in a large population-based randomized clinical trial (32). Hiroki et al. reported two study results: 1) the prognostic value of low urine pH on upper tract recurrence in non-muscle-invasive bladder cancer (19) and 2) the prognostic value of low urine pH for bladder recurrence in upper tract urothelial carcinoma (33). Similarly, our group recently reported that acidic urine was independently associated with a shortened DFS and OS in patients with upper tract urothelial carcinoma, using a prospectively enrolled registry. The above clinical evidence may be explained by the urogenous contact hypothesis which indicates the increased DNA binding of carcinogens, such as 4-aminobiphenyl in the bladder upon acidic urine conditions (34, 35). Furthermore, our group thought various tumor-promoting cellular events might occur when tumor cells encounter an acidic extracellular environment, which is evidently observed in *in vitro* studies (36, 37). In this study, we extended the clinical significance of acidic urine to patients with bladder cancer undergoing radical cystectomy using a prospectively enrolled systemic database. As shown in **Table 5**, acidic urine (urine pH ≤ 5.5) was a significant risk factor for both DFS and OS.

As the low urine pH group may be the result of several confounding factors, we thoroughly compared the low and high urine pH groups in various aspects (**Tables 3**, **4**) and found that there were no clinical or pathological differences. In addition, as urine pH homeostasis is one of the primary functions of the kidney, urine acidity may reflect the degree of renal function decline, as demonstrated in previous studies (38). In this regard, Cao et al. reported that preoperative renal insufficiency was a prognostic factor in urothelial carcinoma in a meta-analysis and systematic review (7). In our study, there were no significant differences; however, the eGFR tended to be lower in the low urine pH group. Regarding its impact on the prognosis, consistent with a previous study, a low eGFR was independently associated with shortened DFS in our study (**Table 5**). Second, because Hiroki et al. showed the clinical significance of acidic urine in bladder cancer and upper tract urothelial cancer, especially in patients with a smoking history, we compared whether smoking history was a confounding factor. There were no differences in the incidence of ever-smokers between the low and high urine pH groups. As smoking history is a well-known risk factor for bladder cancer (5), and Hiroki et al. reported that acidic urine affects the prognosis in patients with a smoking history (19, 33), we assessed Kaplan–Meier survival curves with the log-rank test in patients with or without a history of smoking. In patients with a smoking history, the acidic urine group showed shortened cancer-specific survival (log-rank *P*=0.042) and borderline significantly shortened OS (log-rank *P*=0.075), while there was no significant association in the non-smoker group (log-rank *P*=0.209 and *P*=0.141, respectively). Collectively, we conclude that urine pH is a powerful and independent prognostic factor in patients with bladder cancer undergoing radical cystectomy. As there were no differences in the clinicopathological characteristics, such as tumor size, grade, T stage, N stage, and histologic variant incidence between the low and high urine pH groups, we hypothesized that there may be additional molecular mechanisms leading to tumor recurrence and poor prognosis, which are not clinically defined in the current clinical practice.

In summary, in patients with acidic urine, a low urinary pH may represent an extracellular acidic environment and trigger aggressive tumor cell growth and proliferation, leading to a high recurrence rate and shortened RFS and OS. This study is meaningful in that low urine pH was found as a significant prognostic factor in advanced patients who underwent cystectomy, unlike the previous study of non-muscle invasive bladder cancer (39). Although several studies have investigated the risk factors of bladder cancer, most of them are pathologic features, and risk stratification is still lacking, thus resulting in a dismal prognosis. Furthermore, although several researchers have demonstrated consistent results of the neutrophil-to-lymphocyte ratio as a serologic prognostic factor (40), there has been no such marker for urine, which directly communicates with tumor cells. Although a prospective study is still needed, our novel approach to analyze the role of urine pH may facilitate the risk stratification of patients with bladder cancer undergoing radical cystectomy.

The study included a well-designed prospective enrolled registry cohort with identical workflow from diagnosis to post-treatment follow-up. As urine pH measurement is a non-invasive technique and can be repeated easily, it has an attractive methodological advantage. However, this study has several limitations. First, measuring spot pH using the dipstick test has a methodological limitation, as 24-hour urine collection may more accurately reflect the pH of the patient’s urine. Second, not every confounding factor affecting urine acidity, such as dietary intake or gastrointestinal ailments, was considered. Third, although urine pH was strongly associated with shortened RFS and OS in this study, a prospective randomized controlled study should be conducted to confirm that acidic urine is a strong risk factor. Fourth, we have shown the clinical outcomes of the acidic and non-acidic urine group, however, we could not concretely analyze the etiology of the induced acidity. Finally, modulating extracellular pH and analyzing invasive metastatic features in *in vitro* and *in vivo* models may provide insights into the molecular mechanisms underlying bladder cancer progression and help develop novel treatment strategies.

This study demonstrated the role of acidic urine as a significant prognostic factor in patients undergoing radical cystectomy. Although a prospective study is required in the future, the non-invasive monitoring of urine pH-based patient risk stratification may provide an attractive clinical benefit if accompanied by a reliable methodology and consistent results. Further studies should be conducted to investigate the molecular mechanisms of acidic urine in aggressive bladder cancer.

## Data availability statement

The datasets presented in this article are not readily available due to restricted access only available to the department of urology, Seoul national university hospital. Requests to access the datasets should be directed to JH, jhhan2013@gmail.com.

## Ethics statement

This study was approved by the Institutional Review Board of the Seoul National University Hospital (IRB no. 2205-118-1327). The requirement for informed consent was waived owing to the retrospective study design.

## Author contributions

JK had full access to all study data and takes responsibility for the integrity of the data and accuracy of the data analysis. Study concept and design: JK. Acquisition, analysis, or interpretation of data: JH and JK. Drafting of the manuscript: JH and JK. Statistical analysis: JH, SJ, and HY. Administrative, technical, or material support: JH, CJ, CK, and JK. All authors contributed to the article and approved the submitted version.

## Conflict of interest 

The authors declare that the research was conducted in theabsence of any commercial or financial relationships that couldbe construed as a potential conflict of interest.

## Publisher’s note

All claims expressed in this article are solely those of the authors and do not necessarily represent those of their affiliated organizations, or those of the publisher, the editors and the reviewers. Any product that may be evaluated in this article, or claim that may be made by its manufacturer, is not guaranteed or endorsed by the publisher.

## Supplementary material

Supplementary Figure 1 Urine pH distribution of patients included in this study.
