# Supplementary figures and images for: Acidic urine is associated with poor prognosis in patients with bladder cancer undergoing radical cystectomy
Source: Front Oncol. 2022 Aug 26;12:964571. doi: 10.3389/fonc.2022.964571 (PMC9459327; doi:10.3389/fonc.2022.964571)

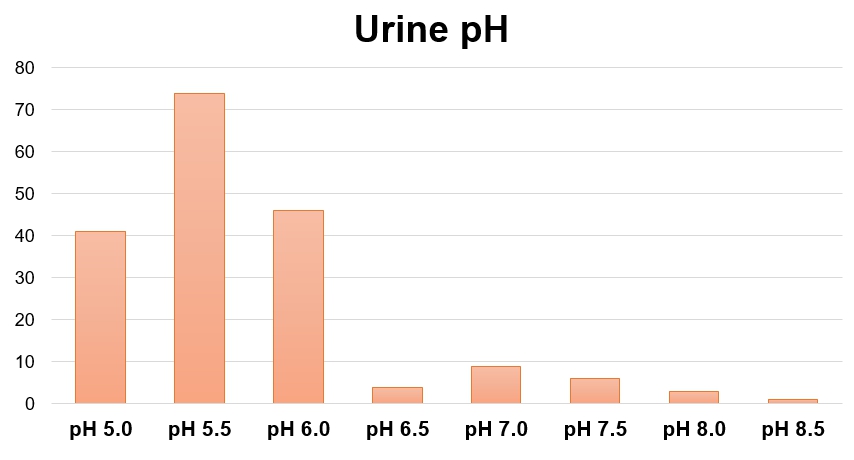

Supplement: Supplementary Figure 1 — Urine pH distribution of patients included in this study. [file Image_1.jpeg]
