# Supplementary material for: Acidic urine is associated with poor prognosis in patients with bladder cancer undergoing radical cystectomy
Source: Front Oncol. 2022 Aug 26;12:964571. doi: 10.3389/fonc.2022.964571 (PMC9459327; doi:10.3389/fonc.2022.964571)
Supplement: Supplementary file 2 [file Table_1.docx]

Supplementary Material

# Supplementary Tables

## Supplementary Table 1. Comparison between low and high Urine pH groups in significant albuminuria patients

|  | Significant Albuminuria (+) | | |
| --- | --- | --- | --- |
|  | Urine pH ≤5.5  (n=37) | Urine pH >5.5  (n=68) | *P*-value |
| Age (years) (mean ± SD) | 71.43±8.81 | 68.74±11.20 | 0.178 |
| Sex (Male) (n, %) | 31 (83.8) | 51 (75.0) | 0.299 |
| BMI (kg/m^2^) (mean ± SD) | 23.96±3.73 | 23.37±4.51 | 0.496 |
| HTN (n, %) | 27 (73.0) | 35 (51.5) | 0.032 |
| DM (n, %) | 13 (35.1) | 18 (26.5) | 0.352 |
| Creatinine (mg/dL) | 1.15±0.37 | 1.02±0.38 | 0.655 |
| eGFR (mL/min/1.73 m^2^) | 66.68±19.23 | 77.20±26.63 | 0.037 |
| Tumor size (cm) (mean ± SD) | 3.68±3.07 | 6.01±4.00 | 0.006 |
| T stage (n, %) |  |  | 0.959 |
| T1-2 | 24 (66.7) | 45 (67.2) |  |
| T3-4 | 12 (33.3) | 22 (32.8) |  |
| N stage |  |  | 0.089 |
| N0 | 30 (88.2) | 47 (73.4) |  |
| N1-2 | 4 (11.8) | 17 (26.6) |  |
| Lymph node density (%) | 1.74±5.67 | 7.35±19.03 | 0.032 |
| Perineural invasion (n, %) | 4 (10.8) | 12 (17.6) | 0.532 |
| Carcinoma in situ (n, %) | 13 (35.1) | 21 (30.9) | 0.805 |
| Lymphovascular invasion (n, %) | 9 (24.3) | 18 (26.5) | 1.000 |
| Venous invasion (n, %) | 2 (5.4) | 5 (7.4) | 0.485 |
| Lymphatic invasion (n, %) | 9 (24.3) | 17 (25.0) | 0.906 |
| Histologic variant (n, %) | 10 (27.0) | 11 (16.2) | 0.252 |
| Neo-adjuvant chemotherapy (n, %) | 7 (18.9) | 16 (23.5) | 0.585 |
| Adjuvant chemotherapy (n, %) | 4 (10.8) | 14 (20.6) | 0.204 |
| Recurrence (n, %) | 10 (27.0) | 20 (29.4) | 0.796 |
| Death (n, %) | 3 (8.3) | 8 (11.8) | 0.588 |

BMI: body mass index, eGFR: estimated glomerular filtration rate
